# Inference of SNP-Gene Regulatory Networks by Integrating Gene Expressions and Genetic Perturbations

**DOI:** 10.1155/2014/629697

**Published:** 2014-06-09

**Authors:** Dong-Chul Kim, Jiao Wang, Chunyu Liu, Jean Gao

**Affiliations:** ^1^Department of Computer Science and Engineering, University of Texas at Arlington, Arlington, TX 76019, USA; ^2^Beijing Genomics Institution at Wuhan, Wuhan 430075, China; ^3^Department of Psychiatry, University of Illinois at Chicago, Chicago, IL 66012, USA

## Abstract

In order to elucidate the overall relationships between gene expressions and genetic perturbations, we propose a network inference method to infer gene regulatory network where single nucleotide polymorphism (SNP) is involved as a regulator of genes. In the most of the network inferences named as SNP-gene regulatory network (SGRN) inference, pairs of SNP-gene are given by separately performing expression quantitative trait loci (eQTL) mappings. In this paper, we propose a SGRN inference method without predefined eQTL information assuming a gene is regulated by a single SNP at most. To evaluate the performance, the proposed method was applied to random data generated from synthetic networks and parameters. There are three main contributions. First, the proposed method provides both the gene regulatory inference and the eQTL identification. Second, the experimental results demonstrated that integration of multiple methods can produce competitive performances. Lastly, the proposed method was also applied to psychiatric disorder data in order to explore how the method works with real data.

## 1. Introduction

In order to understand more accurate causal relationships between a complex disease and genetic variations, we need to consider how the genotypic perturbations affect expression phenotypes that are potentially associated with a target disease. In other words, it is more crucial to look at the overall mechanisms considering a series of three factors, which include genetic variations, altering gene regulations, and caused diseases rather than partial mappings between them. Therefore it is important to evaluate how genetic perturbations affect genes on regulatory networks that are associated with a target disease phenotype. In practice, when biological networks are inferred with high throughput data, we have to consider not only the relationships among genes but also how genetic factors such as single nucleotide polymorphism (SNP) and copy number variation (CNV) can affect genes in gene regulatory network (GRN). Over the last decade, research for mapping genotype to expression phenotype or disease phenotype such as expression quantitative trait loci (eQTL) study and genome wide association study have been actively performed [1]. However, we are now required to do a network-based analysis with genotype data and gene expression because it is more effective in discovering underlying biological process from genotype to phenotype. In doing so, the analysis of SNP-gene regulatory networks (SGRN) will provide more definite relationships of genotypic causes and phenotypic effects so that it will facilitate prognosis and drug designs for therapies.

In this paper we propose a SGRN inference method. In order to identify regulatory interactions among genes, quite a number of network inference methods have been developed by using gene expression data such as gene microarray. Those methods can be generally classified into different theoretical categories: Boolean networks [2, 3], mutual information [4, 5], Bayesian networks (BN) [6, 7], and regression [8, 9]. As each method has its own advantages and limitations under different assumptions and network models such as acyclic or cyclic network and directed or undirected network, there should be trade-offs in inferences given different target network structure and applications [10]. For example, the MI-based approach is very simple and fast so that it can build a large scale network (e.g., genome wide scale) but it cannot estimate direction of edges. It produces worse performance than other approaches in detecting linear cascading structures [10]. The BN-based inference is limited to imply only acyclic network with high computational cost while the regression-based approach supports both directed and cyclic network, which are assumed in SGRN. In addition to directed network model, it should be considered that SGRN is different from conventional GRN inference. In SGRN inference, a gene can be regulated by SNPs as well as other genes, but SNPs are assumed to not be regulated by other SNPs. That is, a SNP cannot be a child node in the network.

Recently, a number of approaches have been suggested to infer SGRNs integrating genetic variation and gene expression data. Kim et al. [11] considered genetic perturbations, gene expression, and disease phenotypes together to find the causal genes to a disease. The electric circuit approach and heuristic search were used to infer SGRN where causal genes are mapped to SNP in the preliminary step before network inference. Keurentjes et al. [12] built a SNP-gene network associated with a particular phenotype, but this method also performed eQTL mapping (SNP-gene) to define the candidate regulator genes before genetic network construction. In addition, Kim and Xing [13] used lasso regression considering the case that a SNP is weakly associated with highly correlated multiple traits rather than a single trait. Chen et al. [14] focused on identifying which pathway among those already known pathways was more likely to be affected by changes of genotype and gene expression rather than inferring a new pathway. The related works we especially noted are the methods that are based on structural equation modeling (SEM) [15–18]. SEM allows us to not only incorporate eQTL information to gene expression in a single model but also identify eQTL simultaneously. However, Logsdon and Mezey [17] assumed that every gene has at least one eQTL, and eQTL mapping was performed by preprocessing but not in a network inference step. Cai et al. [18] introduced sparsity-aware maximum likelihood (SML), which can be potentially extended for eQTL identification. However, SNP-gene pairs were still given in evaluations and implementations of the SML algorithm.

In this paper, we proposed a novel method to infer SGRN where both eQTL identification and SGRN inference are performed simultaneously given a set of gene expression and genotype data without assuming eQTLs are known. The proposed method is based on SEM and multiple steps of edge filtering such as elastic net regression and iterative adaptive lasso. Basically SEM is a regression-based model which is likely to select as many variables causing an overfitting, so the sparsity is enforced by lasso (*l*_1_-regularized least square estimation) considering the sparsity of biological network. Initial weights of edges are estimated by ridge regression [19] and elastic net regression [20], and then the second step is to identify final eQTLs from candidate SNPs selected in the first steps. In the last step, the final network is constructed by iterative adaptive lasso. The first two steps are to fix SNPs before selecting genes. In the third step, edges are selected by iteratively giving more penalties to the edge whose weight is relatively low until network structure is converged.

To evaluate the method, we explore the performance with a simulated data set, that is, generated from random networks with different number of samples and nodes and expected number of edges per node. The result shows that the method can achieve a high detection rate of true edges with low false discovery rate without eQTL information. In addition, to explore the performance in real expression phenotype and SNP data, the method was applied to the psychiatric disorder data. After genes and SNPs were selected from related Genome-Wide Association Study (GWAS), it was tested how the method identify true positive edges between genes and SNPs without eQTL information.

## 2. Method

### 2.1. Problem Definitions

We define the problem and notations here. Let *Y* ∈ *R* ^*M*_*g*_×*N*^ denote the matrix of gene expression levels of *M*_*g*_ genes and *N* samples where a row vector **y**_*i*_ = {*y*_*i*1_,…, *y*_*iN*_} is observed expression level of *i*th gene. *X* is *M*_*s*_ × *N* matrix to denote genotypes of individuals, where *x*_*ij*_ ∈ {1,2, 3} represents the number of minor alleles of *i*th SNP of *j*th sample as an element of matrix *X* supposing that the number of minor alleles should be zero, one, or two in real data. So, *x*_*ij*_ represents a relative quantity of minor alleles of samples. As a gene can be regulated by other genes and genetic variations (SNPs), we define SEM as

(1) $$\begin{array}{c} y_{i}=b_{i}Y+f_{i}X+\mu_{i}+\varepsilon_{i}, \end{array}$$

where **b**_*i*_ denotes *i*th row vector of square matrix *B* ∈ *R* ^*M*_*g*_×*M*_*g*_^; **f**_*i*_ denotes *i*th row vector of square matrix *F* ∈ *R* ^*M*_*g*_×*M*_*s*_^; *μ*_*i*_ is a model bias; and *ε*_*i*_ is a residual modeled as zero-mean Gaussian with a variance *σ* ^2^. As we assume there is no self-regulation (self-loop edge), *b*_*ii*_ = 0, ∀*i* = 1,…, *M*_*g*_, where *b*_*ii*_ denotes *i*th element of **b**_*i*_. The parameters of **b**_*i*_ and **f**_*i*_ decide the network structure defining the weight of regulation from every possible gene and SNP to a target gene *i*. For example, if there is no regulation relationship (directed edge) from *j*th gene to *i*th gene, *b*_*ij*_ is set to zero. Similarly *f*_*ij*_ has nonzero value as a weight of regulation from *j*th SNP to *i*th gene if *j*th SNP is identified as an eQTL for *i*th gene. It is assumed that each gene has at least one eQTL but it is unknown which SNP among a given set of SNPs is an eQTL for a target gene. Our goal in this model is to find *B* and *F* that best fit to observed gene expression and genotype data. To make the problem simpler, we remove *μ*_*i*_ from (1) by applying mean centering for row vectors **y**_*i*_ and **x**_*i*_ to have zero mean. The goal is to find **b**_*i*_ and **f**_*i*_ that minimize a residual *ε*_*i*_, so (1) can be expressed in a least square minimization problem as

(2) $$\begin{array}{c} \underset{b_{i},f_{i}}{\arg\;\min}{||y_{i}-b_{i}Y-f_{i}X||}_{2}^{2}. \end{array}$$

However, regression tends to select as many genes and SNPs as possible to explain the expression level of target gene *i*. To avoid the overfitting, sparse regression methods such as ridge regression, elastic net, and lasso are used.

### 2.2. The Algorithm

The method we propose is based on *l*_1_-regularized linear regression known as lasso [21] that yields a sparsity of variable selection. The algorithm consists of 3 steps, (i)* elastic net*, (ii)* lasso*, and (iii)* iterative adaptive lasso*. The first two steps are to decide *F* where SNPs are selected but their coefficients can be changed in the third step. Then, *B* is finalized by iterative adaptive lasso in the last step.

#### 2.2.1. Ridge Regression (Step 1-1)

In ridge regression, the coefficient values of irrelevant SNPs and genes to a target gene shrink to zero (but not exactly zero) while those of eQTLs and regulator genes of a target gene tend to be higher. Ridge regression of (2) is defined as

(3) $$\begin{array}{c} \underset{b_{i},f_{i}}{\arg\;\min}{||y_{i}-b_{i}Y-f_{i}X||}_{2}^{2}+\lambda_{1}{||b_{i}||}_{2}^{2}+\lambda_{2}{||f_{i}||}_{2}^{2}. \end{array}$$

Given penalty weights, *λ*_1_ and *λ*_2_, the optimal **b**_*i*_ and **f**_*i*_ can be obtained by closed form solution given by

(4) $$\begin{array}{c} f_{i}=\left(y_{i}-b_{i}Y\right)X^{T}{\left(XX^{T}+\lambda_{2}I\right)}^{-1}, \end{array}$$

(5) $$\begin{array}{c} b_{i}=\left(y_{i}-f_{i}X\right)Y^{T}{\left(YY^{T}+\lambda_{1}I\right)}^{-1}. \end{array}$$

Replacing (5) for **b**_*i*_ in (4) yields

(6) $$\begin{array}{c} f_{i}=y_{i}S_{1}{\left(XS_{1}+\lambda_{2}I\right)}^{-1}, \end{array}$$

where

(7) $$\begin{array}{c} S_{1}=X^{T}-Y^{T}{\left(YY^{T}+\lambda_{1}I\right)}^{-1}YX^{T}. \end{array}$$

After calculating **f**_*i*_ first in (6) then (5) can be solved. In this manner, matrices *B* and *F* are estimated by computing each **b**_*i*_ and **f**_*i*_, *i* = 1,…, *M*_*g*_. Parameters *λ*_1_ and *λ*_2_ that decide the degree of sparsity of *B* and *F* are determined by *K*-fold cross-validation. *K* is set to 5 in our experiments.

#### 2.2.2. Elastic Net (Step 1-2)

Note that zero weighted coefficient cannot be recovered back to nonzero in adaptive lasso of Step 3. Therefore, in order to carefully keep only SNPs that are more likely to be true eQTLs in **f**_*i*_, we give *l*_1_-norm penalty to only **f**_*i*_ but not **b**_*i*_ using elastic net defined as

(8) $$\begin{array}{c} \underset{b_{i},f_{i}}{\arg\;\min}{||y_{i}-b_{i}Y-f_{i}X||}_{2}^{2}+\lambda_{1}{||b_{i}||}_{2}^{2}+\lambda_{2}{||f_{i}||}_{1}. \end{array}$$

As the objective function is convex, which guarantees a convergence, *f*_*ij*_ can be optimized by using coordinate descent iteration given parameters, *λ*_1_ and *λ*_2_. To find the optimal **f**_*i*_, the derivative of (8) with respect to *f*_*ij*_ is considered as follows:

(9) $$\begin{array}{c} f_{i}XX_{j}^{T}-y_{i}X_{j}^{T}+b_{i}YX_{j}^{T}+\lambda_{2}\partial_{f_{ij}}{||f_{i}||}_{1}. \end{array}$$

Since the derivative of (8) with respect to **b**_*i*_ is the same as (5), **b**_*i*_ in (9) is substituted with (5), and then (9) is simplified to

(10) $$\begin{array}{c} \left(f_{i(-j)}X_{\left(-j\right)}-y_{i}\right)S_{2}+f_{ij}x_{j}S_{2}-\lambda_{2}\partial_{f_{ij}}{||f_{i}||}_{1}, \end{array}$$

where

(11) $$\begin{array}{c} S_{2}=\left(Y^{T}{\left(YY^{T}+\lambda_{1}I\right)}^{-1}Y-I\right)x_{j}^{T}; \end{array}$$

**f**_*i*(−*j*)_ indicates row vector **f**_*i*_ whose *j*th element is removed, *X*_(−*j*)_ denotes matrix *X* whose *j*th row is removed, and **x**_*j*_ is *j*th row vector of *X*. After defining *C*_*j*_ = (**f**_*i*(−*j*)_ *X*_(−*j*)_ − **y**_*i*_)*S*_2_ and *a*_*j*_ = **x**_*j*_ *S*_2_ in (10), the update rule in the coordinate descent algorithm is written as

(12) $$\begin{array}{c} f_{ij}=\{\begin{array}{cc} \frac{\left(-C_{j}-\lambda_{2}\right)}{a_{j}} & \text{if}\;C_{j}<-\lambda_{2},\\ 0 & \text{if}\;\left|C_{j}\right|\leq \lambda_{2},\\ \frac{\left(-C_{j}+\lambda_{2}\right)}{a_{j}} & \text{if}\;C_{j}>\lambda_{2}. \end{array} \end{array}$$

Algorithm 1 describes the procedures to solve (8) in Step 2. If *f*_*ij*_ is nonzero, *j*th SNP is a candidate eQTL for *i*th gene.

#### 2.2.3. Lasso (Step 2)

In order to finalize a SNP (a single nonzero *f*_*ij*_ of **f**_*i*_) for each gene *i*, we apply lasso to combined matrix of *Y* and *X* as follows:

(13) $$\begin{array}{c} {||y_{i}-h_{i}Z||}_{2}^{2}+\lambda {||h_{i}||}_{1}, \end{array}$$

where

(14) $$\begin{array}{c} Z^{T}=\left[Y_{\left(-i\right)}^{T},X_{\left(-k_{i}^{*}\right)}^{T}\right]. \end{array}$$

*k*_i_* denotes indices of low vectors where *f*_*ij*_ = 0, *j* ∈ *k*_i_*. So, *X*_(−*k*_i_*)_ is a matrix *X* whose *k*_i_* rows are removed. If the number of rows of *X*_(−*k*_i_*)_ is greater than predefined heuristic number *N*_*k*_ (i.e., 5 in our experiments), only top *N*_*k*_ highest *f*_*ij*_ of absolute values of **f**_*i*_ but not all nonzero *f*_*ij*_ are selected for *X*_(−*k*_i_*)_. In Step 2, we iteratively estimate **h**_*i*_, decreasing *λ* from a high value that lets **h**_*i*_ have a zero vector. Regardless of elements of **h**_*i*_ for *Y*_(−*i*)_, we note only which element of **h**_*i*_ for *X*_(−*k*_i_*)_ has a nonzero value first assuming that the corresponding candidate SNP to *h*_*ij*_ is more likely to regulate a target gene *i* if *h*_*ij*_ for a row vector of *X*_(−*k*_i_*)_ has nonzero value earlier than other elements of **h**_*i*_ during *λ* decreases.

#### 2.2.4. Adaptive Lasso (Subroutine of Step 3)

Adaptive lasso is defined as

(15) $$\begin{array}{c} \underset{b_{i},f_{i}}{\arg\;\min}{||y_{i}-b_{i}Y-f_{i}X||}_{2}^{2}+\lambda_{1}{||b_{i}||}_{1,w_{i}^{b}}+\lambda_{2}{||f_{i}||}_{1,w_{i}^{f}}, \end{array}$$

where

(16) $$\begin{array}{c} {||b_{i}||}_{1,w_{i}^{b}}=\overset{N}{\underset{j}{\sum }}\left|b_{ij}\cdot w_{ij}^{b}\right|,\;\;{||f_{i}||}_{1,w_{i}^{f}}=\overset{N}{\underset{j}{\sum }}\left|f_{ij}\cdot w_{ij}^{f}\right|. \end{array}$$

In (16), penalty weights, vectors **w**_*i*_^*b*^ and **w**_*i*_^*f*^, are defined as

(17) $$\begin{array}{c} w_{ij}^{b}={\left|{\hat{b}}_{ij}\right|}^{-\alpha },\;w_{ij}^{f}={\left|{\hat{f}}_{ij}\right|}^{-\beta },\;\forall j=\left\{1,\ldots ,M_{g}\right\}, \end{array}$$

where ${\hat{b}}_{ij}$ and ${\hat{f}}_{ij}$ are estimated in Step 2 that yields a sparsity to **f**_*i*_ but not **b**_*i*_. Zero coefficient of ${\hat{f}}_{i}$ in Step 2 is not considered as an eQTL for gene *i*. So, zero ${\hat{f}}_{ij}$ yields zero *w*_*ij*_^*f*^ in (17), and then if *w*_*ij*_^*f*^ is zero, *f*_*ij*_ will never have nonzero value in adaptive lasso of Step 3 (16). The parameters *α* and *β* decide how much previous estimation such as ${\hat{b}}_{ij}$ or ${\hat{f}}_{ij}$ is reflected to next estimation of *b*_*ij*_ or *f*_*ij*_. Therefore, *f*_*ij*_ that has smaller penalty weight *w*_*ij*_^*f*^ is more likely to have nonzero value. In addition, we consider a special case that *α* and *β* are set to zero supposing that (i) we do not give a penalty weight to *b*_*ij*_ or *f*_*ij*_ by setting *w*_*ij*_^*b*^ or *w*_*ij*_^*f*^ to 1 if ${\hat{b}}_{ij}$ or ${\hat{f}}_{ij}$ is nonzero and (ii) we do not estimate elements of **b**_*i*_ or **f**_*i*_ by setting *w*_*ij*_^*b*^ or *w*_*ij*_^*f*^ to infinity if ${\hat{b}}_{ij}$ or ${\hat{f}}_{ij}$ is zero. The solution is similar to Step 2 in which either **b**_*i*_ or **f**_*i*_ is optimized by coordinate descent algorithm but it is applied to solve both **b**_*i*_ and **f**_*i*_ in Step 3. Derivative of (15) with respect to *b*_*ij*_ yields

(18) biYyjT−yiyjT+fiXyjT+λ1∂bij||bi||1,wib=bijyjyjT+(bi(−j)Y(−j)−yi+fiX)yjT+λ1∂bij||bi||1,wib,

where **b**_*i*(−*j*)_ indicates row vector **b**_*i*_ whose *j*th element is removed and *Y*_(−*j*)_ denotes matrix *Y* whose *j*th row is removed. After setting *C*_*j*_^*b*^ = (**b**_*i*(−*j*)_ *Y*_(−*j*)_ − **y**_*i*_ + **f**_*i*_ *X*)**y**_*j*_^*T*^ and *a*_*j*_^*b*^ = **y**_*j*_ **y**_*j*_^*T*^, the update rule for *b*_*ij*_ is as follows:

(19) bij={(−Cjb−wijb·λ1)ajbif  Cjb<−wijb·λ1,0if  |Cjb|≤wijb·λ1,(−Cjb+wijb·λ1)ajbif  Cjb>wijb·λ1.

We can also estimate *f*_*ij*_ in similar way. After defining *C*_*j*_^*f*^ = (**f**_*i*(−*j*)_ *X*_(−*j*)_ − **y**_*i*_ + **b**_*i*_ *Y*)**x**_*j*_^*T*^ and *a*_*j*_^*f*^ = **x**_*j*_ **x**_*j*_^*T*^, the update rule for *f*_*ij*_ is given as

(20) $$\begin{array}{c} f_{ij}=\{\begin{array}{cc} \frac{\left(-C_{j}^{f}-w_{ij}^{f}\cdot \lambda_{2}\right)}{a_{j}^{f}} & \text{if}\;C_{j}^{f}<-w_{ij}^{f}\cdot \lambda_{2},\\ 0 & \text{if}\;\left|C_{j}^{f}\right|\leq w_{ij}^{f}\cdot \lambda_{2},\\ \frac{\left(-C_{j}^{f}+w_{ij}^{f}\cdot \lambda_{2}\right)}{a_{j}^{f}} & \text{if}\;C_{j}^{f}>w_{ij}^{f}\cdot \lambda_{2}. \end{array} \end{array}$$

When **b**_*i*_ and **f**_*i*_ are updated, updated single element *b*_*ij*_ or *f*_*ij*_ immediately affects updating the next elements. In addition, updating order of elements can be changed since convex objective function is converged in any order of elements to update.  Algorithm 2 shows the optimization procedure of adaptive lasso.

#### 2.2.5. Iterative Adaptive Lasso (Step 3)

Even if **b**_*i*_ and **f**_*i*_ are estimated in Steps 1 and 2, there should be still many false positive edges yet. The primary goal of Steps 1 and 2 is to carefully get rid of only edges that are more unlikely to be true positive edges. So, instead of simply applying adaptive lasso, we developed iterative adaptive lasso to improve the performance of naive adaptive lasso. The motivation of iterative adaptive lasso is that the coefficient value of the variable considerably depends on the value of *α* and *β* which are fixed to 1 and 0.5 in [17, 18], respectively. In iterative adaptive lasso, adaptive lasso is iteratively applied incrementally changing *α* and *β* until there is no more change in the total number of selected edges of *B* and *F* so that more coefficients of irrelevant variables can be shrunk to zero.

Algorithm 3 presents a detailed procedure of iterative adaptive lasso. $\hat{B}$ and $\hat{F}$ estimated in Step 2 are used as arguments. On line 2, *B* and *F* are initialized by ridge regression. Λ_1_^*R*^ is a vector of optimal *λ*_1_ for *B* ^*R*^ estimated by Ridge regression but there is no penalty to *F* ^*R*^ (i.e.  Λ_2_^*R*^ = 0). When *F* ^*R*^ is estimated, only non-zero elements of $\hat{\text{F}}$ that is estimated in Step 2 are updated. On line 5, *B* and *F* are estimated by adaptive lasso in order that elements of *B* are updated by weights of *B* ^*R*^ (i.e. *b*_*ij*_ that has a small value can shrink to zero). Before line 7 starts, ${\hat{\Lambda }}_{1}$ (a vector of ${\hat{\lambda }}_{1}$ for *B* on line 10) is estimated by cross validation of adaptive lasso. On line 7–14, more elements of *B* shrink to zero increasing *α*. The second* while* loop updates *B* until no change in *N*_*e*_(*B*). Once the second* while* loop is terminated, *α* is increased, and then the second loop is performed again. If the second* while* loop is terminated without any change of *N*_*e*_(*B*), the first* while* loop is terminated.

## 3. Results

### 3.1. Simulation Studies

To evaluate the proposed method, we first perform simulations based on randomly generated acyclic networks. The simulation settings are similar to those in [17, 18]. *M* denotes the number of genes and SNPs and is set to 10, 20, and 30. *M* × *N* matrix *B* is initialized to zero matrix where *N* is a sample size; then elements of *B* are randomly selected as directed edges. The selected *b*_*ij*_ has random coefficient value uniformly distributed over 0.5~1 or −0.5~−1. Since we consider a single eQTL per gene (*E*_*s*_ = 1), a single element (*f*_*ii*_) is selected from each row vector (**f**_*i*_). So, *F* is a diagonal matrix. *x*_*ij*_ is randomly set as 1, 2, or 3 with the probabilities 0.25, 0.5, and 0.25, respectively. *Y* is generated by calculating *Y* = (*I* − *B*)^−1^(*FX* + *E*), where *E*_*ij*_ is generated from Gaussian distribution with zero mean and variance 0.01. The number of samples for each network size is *N* = 100, 200, 300, 400, and  500. The number of edges per gene on average is set to *E*_*g*_ = 1, 2, and  3. Given data *Y* and *X*, performances of predicting *B* and *F* are evaluated by comparing true network and inferred network.

**Figure 1:**
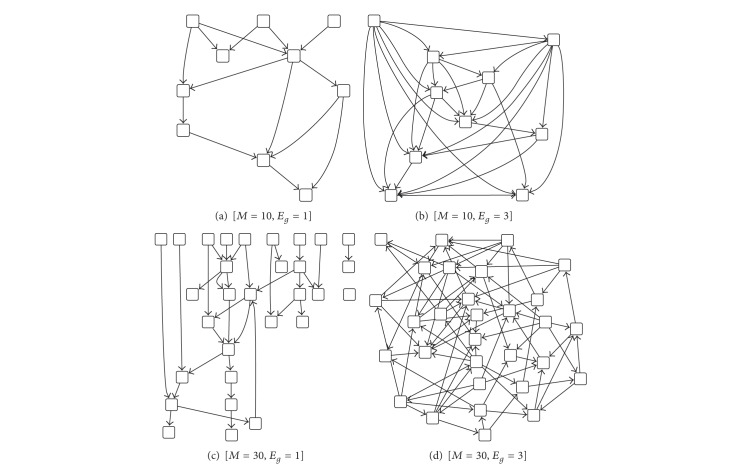
Example of simulated networks with different parameter settings. *M* and *E*_*g*_ indicate the number of genes and expected number of edges per node, respectively.

Figure 1 displays the examples of networks, where SNP nodes are excluded. For the evaluation, true positive (TP), false positive (FP), true negative (TN), and false negative (FN) edges are counted to measure the accuracy criteria such as true positive rate (TPR) and false discovery rate (FDR) that are defined as

- TPR = TP/(TP + FN),
- FDR = FP/(TP + FP).

**Figure 2:**
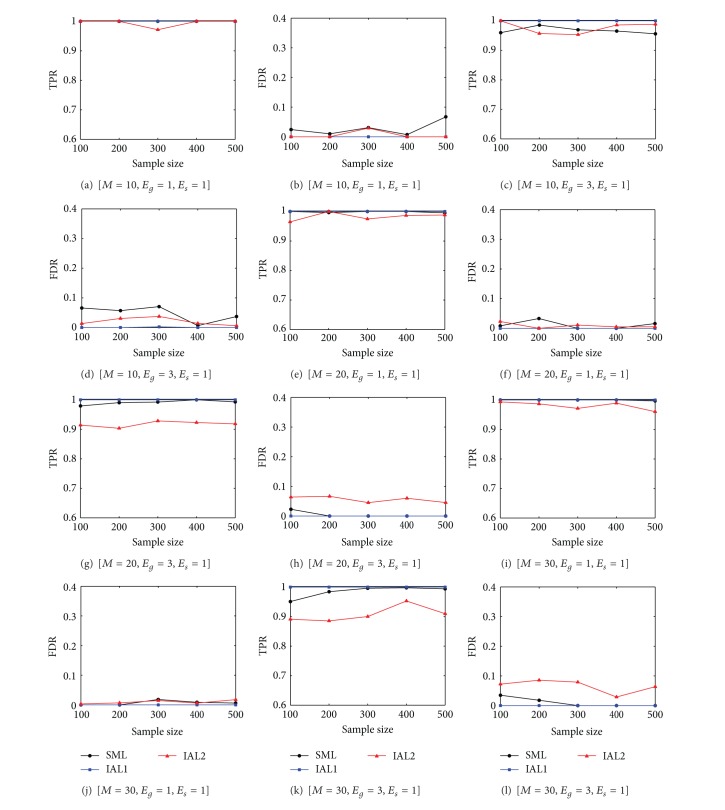
True positive rate and false discovery rate under different numbers of edges and nodes.

In order to evaluate our method, IAL is compared to SML [18]. As SML infers only *B* with known nonzero element indices of *F*, we consider two versions of IAL, IAL without eQTL information and IAL with eQTL information, where Steps 1 and 2 are skipped and only Step 3 is performed with nonzero element index of **f**_*i*_. SML is tested by using the code the author implemented in [18]. The abbreviations of algorithms to compare in Figure 2 and Table 1 are listed below:

- SML: sparsity-aware maximum likelihood algorithm with eQTL information [18],
- IAL1: IAL with eQTL information,
- IAL2: IAL without eQTL information.

Ten replicate simulations are performed and each simulation has a different topology. The results of the different settings (*M* and *E*_*g*_) are displayed in Figure 2. It is shown that IAL1 is superior to SML in all data sets regardless of sample size. We also note that TPR of IAL2 is higher than 0.9 and FDR is less than 0.1 on average in any sample size. It validates that the proposed IAL works very effectively when eQTL is known. In addition, the performance of IAL1 is consistent in different sample sizes while the performance of SML tends to be decreased with small sample size and complicated network (*E*_*g*_ = 3). In network inference, it is known that the performance of inference is very sensitive to the network size and density. In the inference of densely connected and large networks, the computational cost will exponentially increase and the FDR may increase because there are more possible variables that may explain a target node better than true regulators. IAL1 performed consistently in all three different network sizes while the performance of SML is affected by the network size in dense networks (*E*_*g*_ = 3). However, IAL2 shows consistent TPRs and FDRs in all three different network sizes when the network density is normal (*E*_*g*_ = 1) while TPR of IAL2 in Figures 2(g) and 2(k) is lower than Figure 2(c) and also FDR increases in Table 1 when the network size increases in more dense networks (*E*_*g*_ = 2).

The result shows that the performance is better in sparse networks (*E*_*g*_ = 1) than dense networks (*E*_*g*_ = 3) because a complicated structure is more likely to cause false positive edges because of indirect regulations. For example, TPRs in Figures 2(a), 2(e), and 2(i) are much better than in Figures 2(c), 2(g), and 2(k). Similarly FDR is quite increased with *E*_*g*_ = 3 in Figures 2(d), 2(h), and 2(l) compared to the case of *E*_*g*_ = 1 in Figures 2(b), 2(f), and 2(j).

Overall results imply that the proposed IAL1 works perfectly with known *F* in any network size and density. It means that the performance of IAL2 is significantly affected by false positive inference of *F* in steps 1 and 2 because of unknown *F*. More precisely **b**_*i*_ without sparsity in step 2 is more likely to have false positive nonzero elements even though a number of candidate elements of **b**_*i*_ are filtered in step 1. Therefore, the selection of nonzero element of **b**_*i*_ in IAL2 is the most critical part since IAL1 is able to correctly infer *B* only if *F* is given as eQTL information.

### 3.2. Experiments with Psychiatric Disorder Data

**Figure 3:**
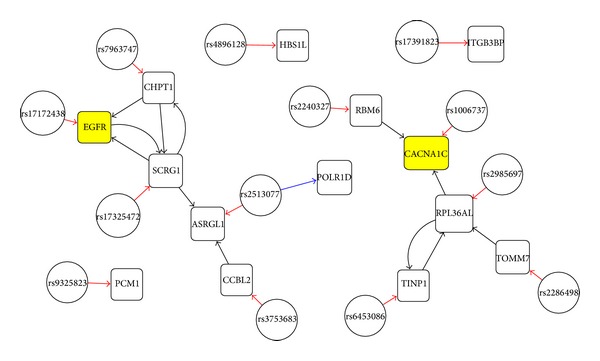
The inferred SGRN with 14 pairs of gene and SNP selected from [22–24].

In this section, the proposed method is applied to real gene expression and genotype data for psychiatric disorder. In the application to real data, we explore the performance of GRN inferences and eQTL identifications through the inferred networks. As far as we know, the proposed method is the first solution to provide both GRN inference and eQTL identification. Thus, the performance comparison with other methods was not performed. The psychiatric disorder data consists of gene expression data of 25833 genes and 852963 SNPs for 131 samples, which were measured from human brain. Since we focus on the network inference but not gene selection, the network construction is performed with a predefined set of genes and SNPs that are selected by preliminary test of multiple sets of genes and eQTLs based on related GWAS for psychiatric disorders. The result of SGRN inference is displayed in Figure 3 where two yellow colored genes, EGFR and CACNA1C, are selected from [23, 24] and the rest of two pairs are from [22]. In applying IAL2 to the data, the weights of *α* and *β* are set to 0.5 instead of 1. Otherwise, *N*_*e*_(**f**_*i*_) tends to be zero. The reason for this is that gene variables are more correlated with their eQTLs because generally eQTLs are independently selected to other genes. In Figure 3, SNP and gene are distinguished by node shape, and a red edge indicates a correct edge from eQTL to corresponding gene. A blue edge represents false positive eQTL mapping. For eQTL identification, one false positive edge appears and thirteen true positive edges are detected (TPR = 0.9286, FDR = 0.0714).

## 4. Discussion

The most difficult part in network inference is to identify directions of edges. In the adjacency matrix *B*, both *B*_*ij*_ and *B*_*ji*_ could have a high coefficient value. In this case, regression-based methods tend to show better performance than MI-based methods because candidate edges are evaluated together in regression-based methods but each edge is independently evaluated to other edges in MI-based methods. Despite the advantage, the regression-based method needs to be integrated with other methods that can provide different information of structure. Another issue to improve in IAL is the computational cost to estimate two different *λs* per each row. Intuitively, a searched optimal *λ* per each row of *B* and *F* should provide a better result but it causes a high computation cost. Lastly, we also assumed that a gene has at least a single eQTL given a set of genes and SNPs, but multiple eQTLs should be considered and a gene may not have any eQTL in practice. Thus, the multiple eQTL of a gene is a future work in SGRN inference.

## 5. Conclusion

In this paper, we proposed a novel network inference method that provides both eQTL identification and network construction of both genes and SNPs. In order to understand gene regulatory mechanisms for a target disease phenotype, the regulatory network inference needs to consider effect of genetic variation and expression phenotype together but not only gene expression data. To achieve the high quality of reliable inference with better TPR and FDR, three different regression skills are integrated. Ridge regression and elastic net are used to remove more likely false positive edges and select eQTL as preliminary steps, and then the finial network is estimated by iterative adaptive lasso removing more false positive edges between genes. Through the experiments with synthetic data, it was demonstrated that IAL1 outperforms SML in SGRN inference and also IAL2 performs eQTL identification effectively. The method was also applied to psychiatric disorder data. Using the genes and eQTLs selected from GWAS of psychiatric disorder, we explored the ability of eQTL identification through inferred SGRN.

## Figures and Tables

**Algorithm 1:**
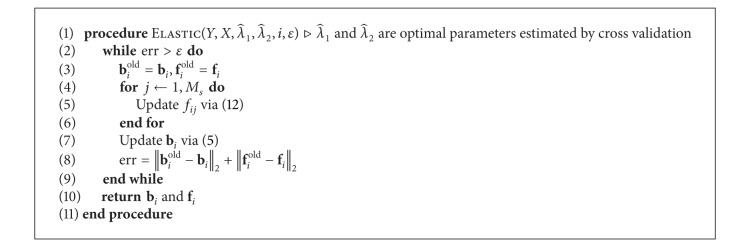
Optimization for *elastic net* in Step 1-2.

**Algorithm 2:**
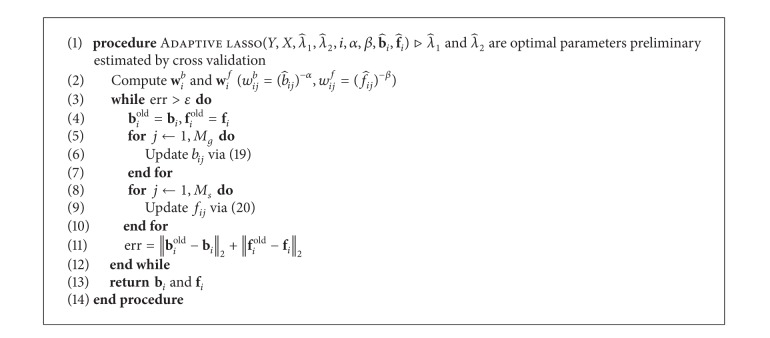
Optimization for adaptive lasso as a subroutine of Step 3.

**Algorithm 3:**
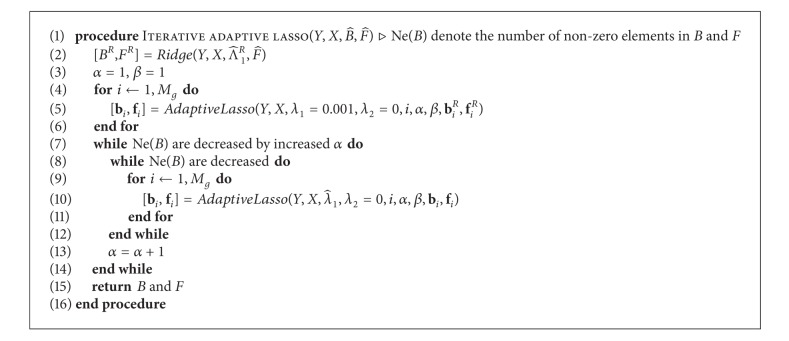
Iterative adaptive lasso in Step 3.

**Table 1:** TPR and FDR of SML, IAL1, and IAL2.

*N*	*M*	TPR			FDR		
		SML	IAL1	IAL2	SML	IAL1	IAL2
100	10	0.9888	1.0000	0.9742	0.0860	0	0.0104
	20	0.9980	1.0000	0.9448	0.0503	0	0.0292
	30	0.9951	1.0000	0.8936	0.0364	0	0.0754

500	10	0.9967	1.0000	1.0000	0.0704	0	0
	20	0.9850	1.0000	0.9436	0.0400	0	0.0369
	30	1.0000	1.0000	0.9128	0.0016	0	0.0562

Expected number of edges per node is *E*_*g*_ = 2 and 10 replicates of random network are used. *N* and *M* indicate the number of samples and genes, respectively.
